# Atrioventricular Block Potentially Associated with Melatonin Supplementation: A Case Report and Systematic Review of Cardiac Rhythm Adverse Events

**DOI:** 10.3390/ph19071069

**Published:** 2026-07-11

**Authors:** Emma Sola, Eva Ramos, Alejandro Romero, Diego Monzón

**Affiliations:** 1 Department of Pharmacology and Toxicology, Faculty of Veterinary Medicine, Complutense University of Madrid, 28040 Madrid, Spain; esola@ucm.es; 2 Cardiovascular Surgery Unit, Gregorio Marañón General University Hospital, Dr. Esquerdo 46, 28007 Madrid, Spain; diego.monzondiaz@gmail.com

**Keywords:** melatonin, arrhythmia, supplementation, atrioventricular block, electrocardiogram

## Abstract

**Background/Objectives:** Melatonin is widely used as a sleep aid and is generally considered safe; moreover, it is well known for its cardioprotective properties. Nonetheless, little is known about the underlying mechanisms of melatonin. Indeed, some potential undesirable cardiovascular effects associated with melatonin supplementation have been observed. **Methods:** We conducted a systematic review to evaluate evidence relevant to a case involving a 63-year-old man who developed ventricular arrhythmia while taking 10 mg of exogenous melatonin daily, exhibiting cardiovascular symptoms, and was then diagnosed with ventricular arrhythmia. After discontinuing melatonin, the patient fully recovered from all cardiovascular symptoms. **Results:** Following withdrawal of melatonin, the patient experienced complete resolution of symptoms and improvement in electrocardiographic findings. The systematic review identified reports describing similar arrhythmic events associated with melatonin supplementation, with symptom resolution or clinical improvement after discontinuation of the supplement. **Conclusions:** Although melatonin is generally regarded as safe, with cardioprotective effects, in this review we discuss possible mechanisms involving melatonin receptors, sleep architecture, pharmacokinetic variability, and genetic predisposition which could be associated with cardiac rhythm and conduction disturbances. Herein, we highlight the necessity for further studies to identify risk factors and underlying mechanisms and guide the safe use of this molecule.

## 1. Introduction

Melatonin is a methoxyindole primarily synthesized and released from the pineal gland under the suprachiasmatic nuclei influence. It plays a crucial role in regulating the body’s circadian rhythm and sleep–wake cycle by transmitting signals relating to darkness, thereby controlling many physiological processes [1]. Although melatonin is commonly regarded as a safe sleep aid, the cardiovascular literature depicts a complex and context-dependent profile. Recent evidence suggests that melatonin may exert cardioprotective effects through antioxidant, anti-inflammatory, anti-apoptotic, endothelial, autonomic, and mitochondrial mechanisms, with potential benefits in ischemia/reperfusion injury, cardiomyopathy, heart failure, vascular dysfunction, and treatment-related cardiotoxicity [2,3,4,5]. Therefore, the present case report and systematic review should not be interpreted as a general statement against melatonin, but rather as an attempt to characterize rare and unexpected cardiovascular events that may occur in selected susceptible individuals. This balanced interpretation is clinically relevant because a compound may show protective properties in experimental or specific clinical settings while still being associated with adverse rhythm or conduction abnormalities under particular biological, pharmacokinetic, genetic, or comorbidity-related conditions.

Over the past several decades, synthetic melatonin has become widely available as an over-the-counter sleep aid and is now commonly used in the management of insomnia.

Despite its reputation as a relatively safe drug, some studies have reported side effects involving the cardiovascular system [6]. Of particular concern is the potential for melatonin-induced cardiac arrhythmias, an infrequent phenomenon underestimated by clinicians. Symptomatic premature ventricular contractions (PVCs) are relatively common in the general population, and are mainly initiated in the ventricular outflow tract [7]. These PVCs are primarily caused by triggered activity and can disturb patients, potentially leading to tachycardia cardiomyopathy [8].

Paradoxically, while melatonin is expected to protect against arrhythmias due to its role in alleviating sleep problems, some reports suggest a strong connection between melatonin administration and the incidence of PVCs from the outflow tract [7]. As outflow tract arrhythmias represent more than 10% of overall referrals for electrophysiological studies, the identification of pharmacological agents that can mediate outflow tract PVC generation is crucial [8,9].

In this systematic review, we analyze the evidence, available in the literature, regarding undesirable cardiovascular effects associated with melatonin supplementation. We also describe a case report of a healthy adult male who developed cardiovascular symptoms and was subsequently diagnosed with melatonin-induced ventricular arrhythmia after daily intake of 10 mg of exogenous melatonin. And in line with some of the evidence found in the literature, after discontinuing melatonin, the patient fully recovered from all cardiovascular symptoms. This case highlights the importance of considering melatonin as a potential trigger for cardiac arrhythmias, even in patients with structurally normal hearts. This approach may also prove safer and more cost-effective than treating the condition with antiarrhythmic medication or catheter ablation.

## 2. Results

### 2.1. Selection and Identification of Relevant Studies

Following both automated and manual screening of the selected databases, 76 records were initially identified. After removing 7 duplicate entries, 69 records remained for further evaluation. Of these, 10 were deemed irrelevant, and 22 were identified as in vivo or in vitro studies. In total, 37 records were assessed in full for eligibility, and 35 were excluded because they did not meet the inclusion criteria or were unrelated to the research topic.

**Figure 1:**
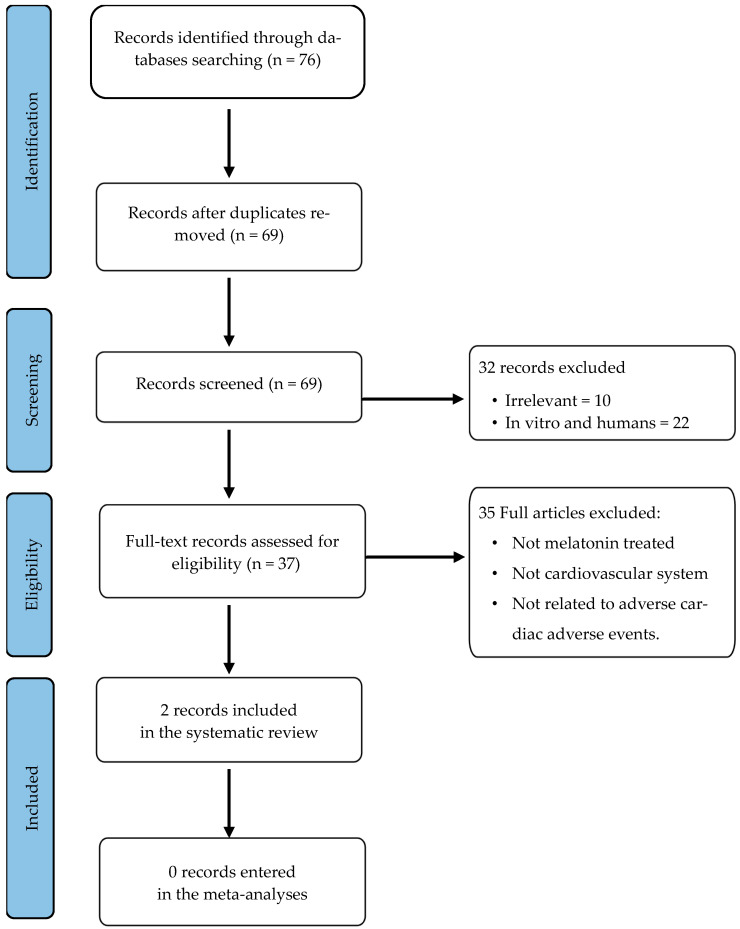
PRISMA flow diagram.

Ultimately, only two studies met the final criteria and were included in the final analysis. A detailed flow diagram of the selection process is represented in Figure 1.

A meta-analysis was not achievable, as only three heterogeneous reports were available; given this limited number of cases, the possibility of publication bias and selective reporting cannot be excluded.

### 2.2. Characteristics of Studies

Table 1 summarizes the more relevant features of the three studies included in this review. Whereas melatonin oral administration was consistent in both studies, no significant differences were noted in the appearance of symptoms, while dosing was quite different: 1 and 10 mg/daily.

Symptomatic premature ventricular contractions (PVCs) are relatively common in the general population, with the majority originating in the ventricular outflow tract [7]. These PVCs are primarily caused by triggered activity and can be disturbing to patients, potentially leading to tachycardic cardiomyopathy.

Paradoxically, while melatonin is expected to protect against arrhythmias due to its role in alleviating sleep problems, some reports suggest a clear association between melatonin use and the occurrence of PVCs from the outflow tract. As outflow tract arrhythmias represent more than 10% of overall referrals for electrophysiological studies, identifying pharmacological agents that can mediate outflow tract PVC generation is crucial [9].

### 2.3. Summary of Cardiovascular Adverse Effects Reported

In both cases, the adverse effects reported are very similar; a potential association between melatonin and idiopathic ventricular arrhythmias is described. In some cases, there are also palpitations and non-sustained ventricular tachycardia (nsVT), with a complete remission or a marked reduction in symptoms when melatonin is discontinued (Table 1). It would be interesting for future studies to apply formal causality assessment tools such as the Naranjo algorithm or WHO-UMC criteria.

## 3. Case Report

### Case Presentation

**Figure 2:**
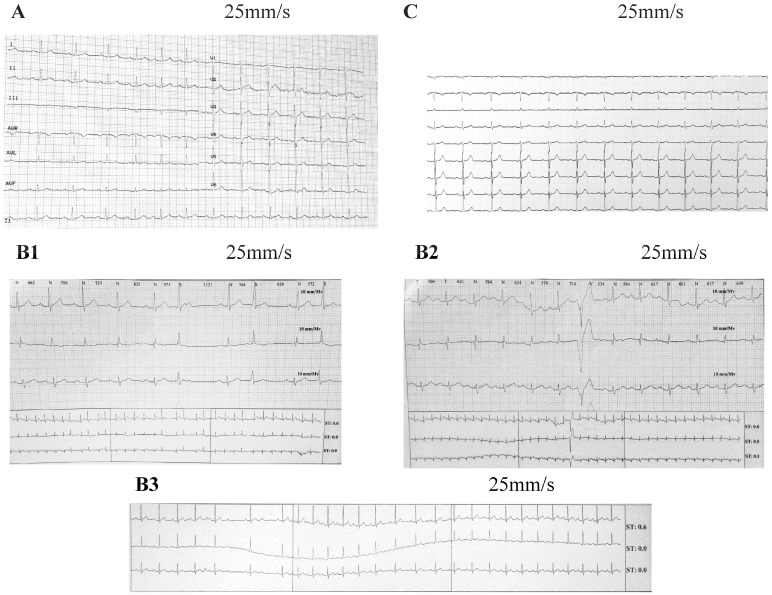
(**A**) Normal ECG in sinus rhythm. (**B1**) Supraventricular ectopy, (**B2**) ventricular ectopy, and (**B3**) second-degree AVB. (**C**) ECG in sinus rhythm after melatonin withdrawal.

A 63-year-old man with dyslipidemia and no other significant medical history presented for evaluation. His usual treatment included 10 mg of melatonin every 24 h. During follow-up for his dyslipidemia, laboratory tests showed no significant abnormalities except for hypercholesterolemia. An electrocardiogram (ECG) was normal, showing sinus rhythm with a PR interval of 200 milliseconds (ms), QRS duration of 92 ms, corrected QT interval (QTc) of 366 ms, and no repolarization abnormalities (Figure 2A). A transthoracic echocardiogram showed normal biventricular function and no valvular disease.

Two months later, he was referred to the Cardiology Department due to a history of palpitations. A Holter monitor was performed that same month, showing sinus rhythm with an average heart rate of 82 bpm, low-density supraventricular ectopy (4 per hour) (Figure 2(B1)), low-density ventricular ectopy (6 per hour) (Figure 2(B2)), and a run of four ventricular ectopic beats. The recording did not show any pauses longer than 3 s. Still, it did reveal first-degree atrioventricular block (AVB) with a maximum PR interval of 298 milliseconds, second-degree AVB, and Mobitz type I and II during the night with a maximum pause of 2200 milliseconds (Figure 2(B3)). As no identifiable cardiac cause could explain the conduction abnormality, and the patient reported that his palpitations had begun after initiating melatonin therapy, melatonin was discontinued and a repeat cardiac evaluation was performed.

Four months after the follow-up, blood tests were repeated and showed no relevant abnormalities. A new Holter monitor revealed that the patient remained in sinus rhythm with an average heart rate of 77 bpm. Supraventricular ectopy was occasional, ventricular ectopy was low-density (2 per hour), and the maximum PR interval was 260 ms with no episodes of second-degree AVB. The patient reported a clear reduction in arrhythmic symptoms. An exercise stress test was performed and was clinically and electrocardiographically negative for ischemia, with a normal blood pressure response and no arrhythmias, achieving 9 METs (functional class I).

At the most recent follow-up, the patient remains free of palpitations following discontinuation of melatonin. Electrocardiography demonstrated normal sinus rhythm, with a baseline PR interval of 160 ms (Figure 2C). To our knowledge, this is the first documented case in which there is a suspicion of second-degree AVB as a consequence of melatonin use.

## 4. Discussion

To our knowledge, this is the first report describing a possible association between melatonin use and the development of second-degree AVB. While melatonin is widely used as a sleep aid and has been studied for its potential cardioprotective properties, emerging clinical reports have documented cases where melatonin supplementation appears to trigger cardiac rhythm disturbances—mostly heart palpitations and arrhythmias—in patients taking melatonin supplements, with symptoms resolving after discontinuation. The small number of identified record limits the strength of the evidence and so it should be interpreted with caution.

AVB encompasses a wide range of conduction disturbances between the atria and the ventricles, and its etiology varies according to the type and anatomical level of the block. The underlying mechanisms can be broadly classified into degenerative, ischemic, pharmacologic, infectious, infiltrative, metabolic, congenital, and postoperative causes [10].

The underlying etiology of AVB often correlates with the site and severity of the conduction abnormality. Classification by AVB type can aid in clinical decision-making and risk stratification. First-degree AVB typically results from delayed conduction within the AV node. It is often benign and asymptomatic. Second-degree AVB—Mobitz Type I (Wenckebach) is usually due to a functional block within the AV node and is often reversible. In 2:1 AVB, every other atrial impulse fails to conduct. It may be due to AV nodal or infranodal disease. High-degree (advanced) AVB is characterized by two or more consecutive non-conducted P waves. In third-degree (complete) AVB, there is no conduction from the atria to the ventricles [10].

Melatonin has not been associated to date with any deleterious effects on cardiac conduction, and no scientific evidence has been found linking melatonin administration to the occurrence of AVB. However, it remains to be determined whether, like other antiarrhythmic drugs, melatonin could trigger the onset of AVB in predisposed individuals.

### 4.1. Observational Studies and Case Reports

A significant clinical report presented two patients with structurally normal hearts who developed symptomatic premature ventricular contractions (PVCs) while taking melatonin supplements [7]. Both patients were elderly men taking 1 mg of melatonin daily for sleep problems. Importantly, discontinuation of melatonin led to complete cessation of PVCs and symptoms in both cases, providing compelling evidence of a causal relationship [7]. Another documented case involved a 55-year-old male with frequent PVCs who had been taking melatonin (initially 5 mg, increased to 10 mg) before bedtime for a year. When melatonin was discontinued, follow-up Holter monitoring showed a progressive decrease in arrhythmia burden from 10% to less than 2% over four months [9]. Beyond individual case reports, a clinical study involving hypertensive patients, which were well-controlled on a calcium channel blocker (nifedipine), found that evening administration of melatonin induced an increase in blood pressure and heart rate throughout the whole day (24 h period) [11], observing that the heart rate tended to be higher for the entire 24 h period, although the increase was statistically significant and clinically important only during the morning and the afternoon hours [11].

In addition to cases of premature ventricular contractions temporally associated with melatonin supplementation [7], other case reports have described rhythm or conduction-related findings after melatonin exposure. Alawad et al. [12] reported symptomatic bradycardia in an otherwise healthy 22-year-old male who presented with palpitations and dizziness after ingesting 20 mg of melatonin; the bradycardia resolved spontaneously after clinical observation. Hassan et al. [13] described a Brugada type 2 electrocardiographic pattern in the context of melatonin exposure and alcohol intoxication, emphasizing that melatonin overdose may contribute to reversible Brugada-like patterns after exclusion of other causes. These reports should not be interpreted as definitive proof of causality, but they broaden the clinical spectrum of potential rhythm and conduction disturbances temporally associated with melatonin intake. They also reinforce the need to document supplement use in patients presenting with otherwise unexplained palpitations, bradyarrhythmias, ventricular ectopy, conduction abnormalities, or transient electrocardiographic patterns.

Furthermore, consistent with the studies selected in the systematic review, we present a case report, where a healthy male adult exhibited cardiovascular symptoms and was subsequently diagnosed with melatonin-induced atrioventricular block and ventricular arrhythmias after daily intake of 10 mg of exogenous melatonin. This case highlights the importance of considering melatonin as a potential trigger for cardiac arrhythmias, even in patients with structurally normal hearts. This approach also may prove safer and more cost-effective than treating the condition with antiarrhythmic medication or catheter ablation.

### 4.2. Temporal Relationship with Melatonin and Confounders

The temporal relationship between melatonin administration and the adverse event may be related with a potential causal association, because the patient was only treated with a daily dose of 10 mg of melatonin and no other treatments. Additionally, the palpitations reported by the patient appeared from the beginning of administration. Also, the conduction disturbance and ventricular ectopic beats were recorded while the patient was on melatonin treatment and, after its discontinuation, the patient recovered with a normal ECG.

There were no cofounder factors detected, there was no cardiac pathology, the laboratory tests were normal except for hypercholesterolemia, the basal ECG was normal, and the exercise stress test was negative for ischemia, with a normal blood pressure response and no arrhythmias observed.

Therefore, the association with melatonin is supported by the discontinuation course and weakened by the absence of other clear triggers. Regardless, further studies are needed to stablish a causal relationship.

### 4.3. Potential Mechanisms

Several biological mechanisms may explain how melatonin could potentially induce cardiac rhythm disturbances in susceptible individuals. Melatonin has a direct cardiac effect through the melatonin receptors (MT1 and MT2), which have been detected in cardiovascular tissues [7]. Through these G-protein-coupled receptors, melatonin is thought to alter the function of key players in calcium handling within cardiac cells, including L-type calcium channels, ryanodine receptors, and sarcoplasmic/endoplasmic reticulum calcium ATPase [7]. These alterations could potentially create favorable conditions for developing cardiac rhythm disturbances in susceptible individuals. On top of this, recent research challenges the traditionally held view of melatonin as solely antiarrhythmic, with some studies finding that melatonin increases susceptibility to atrial fibrillation when linked to obesity via Akt signaling impairment [14].

It has been reported that melatonin receptors influence cardiac electrophysiology in particular, through the MT2 receptors that are encoded by the MTNR1B gene, which has been linked to cardiovascular homeostasis. In this respect, rs10830963 polymorphisms were related to altered receptor expression and function, elevated melatonin levels, and disrupted circadian rhythms. MTNR1B variations have been associated with myocardial infarction or arrhythmias; however, this connection has not been definitively established [15].

Melatonin may also induce cardiac rhythm disturbances through indirect mechanisms. For instance, melatonin has been observed to paradoxically reduce deeper sleep in some individuals [9]. This alteration in sleep architecture could indirectly promote cardiac rhythm disturbances, as sleep disturbances are known to be related to cardiac rhythm [9]. Another indirect mechanism involves interactions with the sympathetic nervous system. While melatonin generally reduces sympathetic tone, it may, under certain conditions, increase sensitivity to noradrenaline [11]. In hypertensive patients taking calcium channel blockers, melatonin appeared to impair the antihypertensive efficacy of the calcium channel blocker, possibly through competition between the two substances [11].

### 4.4. Contrasting Evidence: Cardioprotective Effects

Interestingly, a significant body of research suggests that melatonin may have cardioprotective and even antiarrhythmic properties in certain contexts, creating an apparent paradox. For example, animal studies have demonstrated that melatonin can reduce the incidence of ventricular tachycardia and ventricular fibrillation by shortening the baseline activation time and repolarization interval and, thereby, improve overall repolarization time [4]. Recent reviews have summarized evidence supporting the beneficial effects of melatonin in cardiovascular disease, mainly through a reduction in oxidative stress and inflammation, preservation of mitochondrial function, modulation of endothelial function, attenuation of myocardial remodeling, and regulation of apoptosis and autophagy [2,3,4]. These mechanisms are biologically relevant because oxidative injury, mitochondrial dysfunction, altered Ca^2+^ homeostasis, gap-junction remodeling, and autonomic imbalance are all involved in arrhythmogenesis and myocardial injury. In this respect, a recent study has also emphasized that melatonin may regulate mitochondrial dynamics and mitophagy, contributing to cardioprotection against ischemia/reperfusion injury, cardiomyopathies, atherosclerosis, and cardiotoxicity in nonclinical models [5].

Importantly, several electrophysiological studies support a potential antiarrhythmic effect of melatonin. In experimental rat hearts, blockade of MT1/MT2 melatonin receptors abolished the antiarrhythmic effect of melatonin and slowed ventricular conduction, suggesting that receptor-dependent signaling may preserve impulse conduction through effects on connexin-43 phosphorylation, resting membrane potential, and inward rectifier potassium current [16]. These findings are relevant because they confirm that melatonin can directly influence cardiac electrophysiology. However, this same biological plausibility also supports the possibility that, in certain predisposed individuals or pathological contexts, melatonin-related electrophysiological modulation could contribute to unexpected rhythm disturbances.

Clinical evidence also supports a potential cardiovascular benefit in selected patient populations. A recent systematic review and meta-analysis in patients with heart failure reported improvements in quality of life and several functional or biochemical parameters after melatonin supplementation, although the available clinical evidence remains limited and heterogeneous [17]. Taken together, these data reinforce the need to avoid an oversimplified interpretation. The present case and systematic review do not negate melatonin’s cardioprotective potential; rather, they highlight a possible “melatonin paradox”, whereby a compound with protective effects in many experimental and clinical contexts may, rarely, be associated with arrhythmic or conduction events in vulnerable individuals. Such vulnerability may be influenced by dose, formulation, timing of administration, sleep architecture, comorbidities, concomitant drugs, obesity-related signaling changes, pharmacokinetic variability, and genetic predisposition.

### 4.5. Risk Factors and Dosage Considerations

The available evidence suggests that several factors may influence the likelihood of experiencing melatonin-induced cardiac effects. While some individuals report palpitations even at low doses, most documented cases involve higher doses, and case reports from the medical literature involve patients taking between 1 and 10 mg daily [7,9]. This emphasizes that not only the dosage but also individual variability in melatonin metabolism and sensitivity appear to play a critical role.

Melatonin bioavailability shows substantial person-to-person variability, with up to a 25-fold difference in the area under the curve following a single dose [7]. This pharmacokinetic variability may explain why some individuals experience cardiac effects while others do not at similar or higher doses.

## 5. Materials and Methods

We conducted the selection and bibliographic analysis following the systematic review methodology outlined in the PRISMA statement, reporting our results according to the Preferred Reporting Items for Systematic Reviews (PRISMA) guidelines, and the review was prospectively registered on PROSPERO (CRD42023427693) [18].

The four authors subsidiarily designed and validated the review protocol. Following PRISMA guidelines, we gathered related studies, assessed the quality and relevance of each study to the selected topic, extracted relevant data, and performed a critical synthesis.

Using the planned methodology, we identified all clinical studies and case reports addressing arrythmias in the same period the patient was treated with melatonin published up to 18 April 2026. The adoption of the PRISMA protocol ensured transparency and reproducibility in both the selection criteria and analytical procedures. The step-by-step methodology, following the PRISMA framework, is detailed below. Additionally, this systematic review was registered in PROSPERO [CRD42023427693].

### 5.1. Search Strategy

The systematic search for the primary reports was carried out in the main repositories of biomedical information: PUBMED Medline (https://pubmed.ncbi.nlm.nih.gov) (accessed up to 18 April 2026), EBSCO (https://www.ebsco.com) (accessed up to 18 April 2026), and the Cochrane Library (https://www.cochranelibrary.com) (accessed up to 18 April 2026). The identification of the relevant literature was conducted employing key terms and Boolean operators combined in the following search algorithm: (“melatonin”[Title/Abstract]) AND (“arrhythmia”[Title/Abstract] OR “atrioventricular block”[Title/Abstract] OR “conduction abnormalities”[Title/Abstract] OR “premature ventricular contractions”[Title/Abstract]).

Medical Subject Headings (MESH) terms were used to ensure the identification of all studies related to the central point of the revision. Moreover, the systematic search was complemented by hand-searching to ensure comprehensive coverage of the available literature.

### 5.2. Inclusion and Exclusion Criteria

Only original human studies published in English in which the patients that developed ventricular arrhythmias were treated with melatonin were included, and review articles were excluded.

### 5.3. Study Selection

We use EndNote to export the results (https://www.myendnoteweb.com), and all duplicate entries were removed. Article selection was based on whether titles and abstracts were pertinent; when necessary, a full-text review was performed. Any discrepancy related to any of the studies about eligibility or inclusion were resolved through consensus among the authors.

### 5.4. Quality Assessment

The quality assessment of the included articles was independently performed, reviewing the study design, because both articles were case reports and quality or risk of bias is not possible to be assessed in clinical studies. For the quality assessment development, the criteria were clarity in the patient case description, temporal relationship between melatonin administration and the adverse event, exclusion of alternate causes or confounding factors, consistency of clinical and electrocardiographic findings, and documentation of the case report after drug withdrawal.

Therefore, due to the nature of the articles and case reports, the quality assessment was focused on the clinical reliability of the association rather than on a formal estimation of the risk of bias.

## 6. Conclusions

This case report and systematic review underscore the need for greater awareness among healthcare professionals regarding the potential cardiovascular adverse effects associated with melatonin use, especially cardiac arrhythmias. While melatonin is generally regarded as safe and is known for its positive impact on sleep and heart health, new findings indicate that it may unexpectedly trigger irregular heart rhythms—such as premature ventricular contractions and AVB—in certain vulnerable individuals.

Medical professionals should stay alert and consider melatonin intake as a potential cause when patients present with unexplained heart rhythm problems or conduction issues, particularly in the absence of structural heart disease. Close monitoring and thorough assessment of patients taking melatonin, especially in higher doses ranging from 1 to 10 mg per day, are advised to quickly detect and address any harmful cardiovascular reactions.

As melatonin continues to gain popularity as an over-the-counter remedy for sleep, more research is needed to better understand its effects on the heart, how it behaves differently in various individuals, and the biological mechanisms behind these effects. Such studies will help promote safer medical practices, ensure more effective use of melatonin, and reduce the risk of heart-related side effects.

## Figures and Tables

**Table 1:** Characteristics of the three in vivo studies included in the two records selected.

	Case 1 [7]	Case 2 [7]	Case 3 [9]
**Age**	72	63	55
**Sex**	Male	Male	Male
**Melatonin dosage**	1 mg/daily	1 mg/daily	10 mg/daily
**Duration**	Unknown	Unknown	1 year
**Symptoms**	Palpitations	Palpitations	Palpitations
**Holter**	2000 multiform PVCs ^1^/24 h	PVCs ^1^, nsVTs ^2^	16–18% PVCs ^1^ of LV ^3^ origin
**Outcome (after** **stopping melatonin)**	Improvement	Improvement	Improvement
**History of cardiovascular disease**	Short episode of paroxysmal SVT ^4^ 34 years earlier	AVRT ^5^, AT ^6^, AF ^7^, AFl ^8^ (successful catheter ablation a year earlier)	Systemic hypertension

^1.^ PVCs: premature ventricular contractions; ^2^ nsVTs: non-sustained ventricular tachycardias; ^3^ LV: left ventricle; ^4^ SVT: supraventricular tachycardia; ^5^ AVRT: atrioventricular re-entry tachycardia; ^6^ AT: atrial tachycardia; ^7^ AF: atrial fibrillation; ^8^ AFl: atrial flutter.

## Data Availability

The original contributions presented in this study are included in the article material. Further inquiries can be directed to the corresponding authors.
